# Supplementary material for: Erythroid Differentiation Regulator 1 Ameliorates Collagen-Induced Arthritis via Activation of Regulatory T Cells
Source: Int J Mol Sci. 2020 Dec 15;21(24):9555. doi: 10.3390/ijms21249555 (PMC7765345; doi:10.3390/ijms21249555)
Supplement: Supplementary file 1 [file ijms-21-09555-s001.pdf]

Supplementary Figures

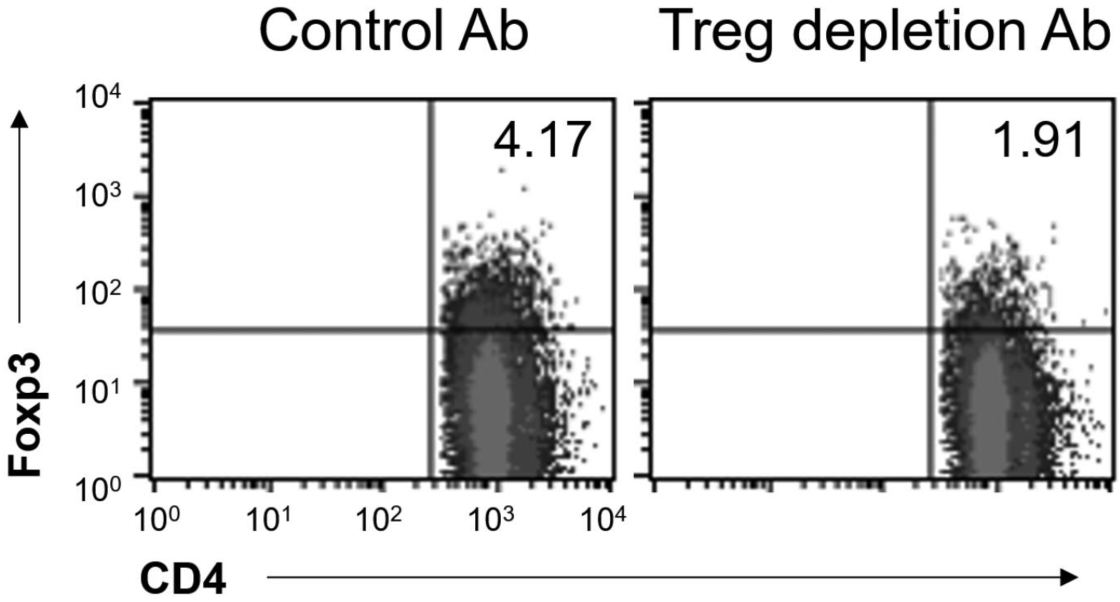

Figure S1. Confirmation of regulatory T (Treg) cell depletion. Treg depletion antibody or isotype control antibody was administrated to collagen-induced arthritis (CIA) mice, and 48h later draining lymph nodes were collected. Treg populations were compared using flow cytometry.

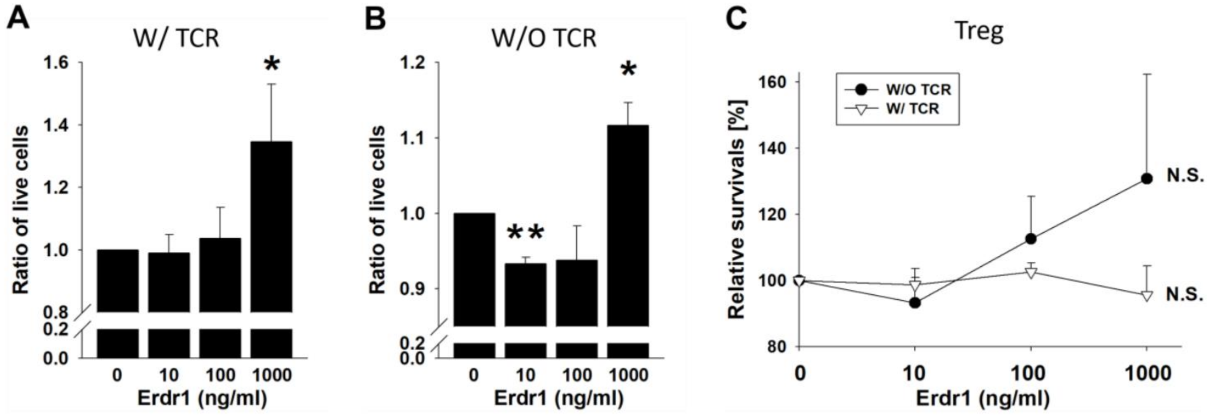

Figure S2. T cell viability. CD4 T cells were treated with various doses of Erythroid differentiation regulator 1 (Erdr1) in the presence (**A**) or absence (**B**) of T cell receptor (TCR) stimulation for 48 h, and cell viabilities were presented as mean ± SD from three independent experiments in triplicate (n = 3). (**C**) Relative survivals of Treg cells (gated on $CD4^{+}$ $+Foxp3^{+)}$ in the absence or presence of TCR stimulation were expressed as mean ± SD from three independent experiments in triplicate (n = 3). Relative cell viability and survival were evaluated based on the percentages of flow cytometric gate for live lymphocytes.* $p < 0.05$.
